# Proteome and Microbiome Mapping of Human Gingival Tissue in Health and Disease

**DOI:** 10.3389/fcimb.2020.588155

**Published:** 2020-10-02

**Authors:** Kai Bao, Xiaofei Li, Lucy Poveda, Weihong Qi, Nathalie Selevsek, Pinar Gumus, Gulnur Emingil, Jonas Grossmann, Patricia I. Diaz, George Hajishengallis, Nagihan Bostanci, Georgios N. Belibasakis

**Affiliations:** ^1^Division of Oral Diseases, Department of Dental Medicine, Karolinska Insitutet, Huddinge, Sweden; ^2^Department of Basic and Translational Sciences, School of Dental Medicine, Philadelphia, PA, United States; ^3^Functional Genomic Centre, ETH Zurich and University of Zurich, Zürich, Switzerland; ^4^Swiss Integrative Center for Human Health, Fribourg, Switzerland; ^5^Department of Periodontology, School of Dentistry, Ege University, Izmir, Turkey; ^6^Department of Oral Biology, University at Buffalo, State University of New York, Buffalo, NY, United States

**Keywords:** tissue proteomic analysis, biofilm, gingiva, periodontitis, microbiome

## Abstract

Efforts to map gingival tissue proteomes and microbiomes have been hampered by lack of sufficient tissue extraction methods. The pressure cycling technology (PCT) is an emerging platform for reproducible tissue homogenisation and improved sequence retrieval coverage. Therefore, we employed PCT to characterise the proteome and microbiome profiles in healthy and diseased gingival tissue. Healthy and diseased contralateral gingival tissue samples (total *n* = 10) were collected from five systemically healthy individuals (51.6 ± 4.3 years) with generalised chronic periodontitis. The tissues were then lysed and digested using a Barocycler, proteins were prepared and submitted for mass spectrometric analysis and microbiome DNA for 16S rRNA profiling analysis. Overall, 1,366 human proteins were quantified (false discovery rate 0.22%), of which 69 proteins were differentially expressed (≥2 peptides and *p* < 0.05, 62 up, 7 down) in periodontally diseased sites, compared to healthy sites. These were primarily extracellular or vesicle-associated proteins, with functions in molecular transport. On the microbiome level, 362 species-level operational taxonomic units were identified. Of those, 14 predominant species accounted for >80% of the total relative abundance, whereas 11 proved to be significantly different between healthy and diseased sites. Among them, *Treponema* sp. HMT253 and *Fusobacterium naviforme* and were associated with disease sites and strongly interacted (*r* > 0.7) with 30 and 6 up-regulated proteins, respectively. Healthy-site associated strains *Streptococcus vestibularis, Veillonella dispar, Selenomonas* sp. HMT478 and *Leptotrichia* sp. HMT417 showed strong negative interactions (*r* < −0.7) with 31, 21, 9, and 18 up-regulated proteins, respectively. In contrast the down-regulated proteins did not show strong interactions with the regulated bacteria. The present study identified the proteomic and intra-tissue microbiome profile of human gingiva by employing a PCT-assisted workflow. This is the first report demonstrating the feasibility to analyse full proteome profiles of gingival tissues in both healthy and disease sites, while deciphering the tissue site-specific microbiome signatures.

## Introduction

As a consequence of the microbial challenge, periodontitis causes destruction of underline connecting tissue, including gingival epithelial layer, which builds a barrier to the external challenge. Thus, microbial invasion of the periodontal tissues may take place during the respective pathological processes (Colombo et al., 2007). In earlier transmission electron microscopy studies, invasion of spirochetes and other microorganisms were evident in the gingival epithelium and connective tissues, especially in patients with acute necrotising gingivitis (Listgarten, 1965; Courtois et al., 1983). This tissue invasive feature is different from that of endocytosis by non-phagocytic host cells, through which bacteria evade phagocytic elimination by the immune system. However, bacterial invasion has traditionally been considered to take place at relatively late stages. Based on this rationale, most periodontal microbiome studies have been focused on the characterisation of biofilms. It is plausible that, at least in part, bacterial invasion is involved in the pathogenesis of periodontitis. Interestingly, one study showed that bacteria could form a biofilm-like structure within the gingival tissue (Baek et al., 2018). Further, 16s rRNA profiling analysis has shown that *Fusobacterium nucleatum* and *Porphyromonas gingivalis* were highly enriched within the tissue compared with the plaque (Baek et al., 2018). Although most available microbiome studies were mainly focused on the bacterial plaque (biofilm), an overall microbiome map directly derived from human gingival tissues is necessary to draw the whole picture for understanding this virulence aspect of periodontal disease.

With the help of mass spectrometry, researchers can identify thousands of proteins for a given sample in a single run (Bostanci and Bao, 2017), which is ideal for delivering a snapshot of protein regulations within the gingival tissue. However, attempts to map gingival tissue proteomes have been hampered by lacking sufficient protein extraction workflows. Bertoldi et al. (2013) reported 13 gingival proteins differentially regulated in diseased sites, compared with their neighboring inter-proximal healthy sites. This included the upregulation of annexin A2, actin cytoplasmic 1, carbonic anhydrase 1 and 2; Ig kappa chain C region and flavinreductase as well as downregulation of 4-3-3 protein sigma and zeta/delta, heat-shock protein beta-1, triosephosphateisomerase, peroxiredoxin-1, fatty acid-binding protein-epidermal, and galectin-7 in pathological tissues. Monari et al. identified 32 different protein spots and elevation of S100A9, 14-3-3 protein zeta/delta, Heat shock protein beta-1 and Galectin-7 in gingival tissues from periodontal patients compared with those from healthy individuals (Monari et al., 2015). Whereas, Yaprak et al. (2018) identified 47 proteins from healthy gingival tissue, including 14-3-3 protein sigma, S100A9 and Galectin-7, which also identified in the works of Bertoldi et al. and Monari et al. Yet, although transcriptomic and proteomic patterns are rarely similar (Wang et al., 2017), transcriptomic analysis of gingival tissues has identified as many as 12,744 expressing genes (Demmer et al., 2008), indicating that there is plenty of space for the improvement for proteomic identification.

A sensitive pressure cycling technology (PCT)-assistant workflow with proven efficiency in gingival tissue disruption (Bao et al., 2019) was used in this study. The present study aimed to concomitantly characterise the gingival tissue proteome and microbiome of systematically healthy individuals with periodontitis, by comparing healthy and diseased sites. Label-free quantitative proteomics and 16SrRNA gene sequencing platforms were applied to dissect the relationship between bacterial abundance and protein regulation among these gingival tissues.

## Results

### Proteome Profiles of Gingival Tissue Samples Cluster Based on Clinical State

The gingival tissue proteome charted in this study derived from 10 gingival tissues (one healthy and one diseased site per individual) obtained from 5 individuals with stage III periodontitis. Prevalence of teeth with one or more sites with probing pocket depth (PPD) > 5 mm and PPD > 5 mm were % 44 ± 5.3 and % 35.6 ± 6.3. Approximately 82% of sites with PPD > 5 mm had bleeding on probing (BOP). The mean PPD and clinical attachment loss (CAL) scores of the sampled diseased sites were significantly higher than the healthy ones [*p* < 0.05, PPD (mm): 2.2 ± 0.8 vs. 7.0 ± 0.7, *p* < 0.001, CAL (mm): O vs. 8.0 ± 0.7, *p* < 0.0001].

**Figure 1:**
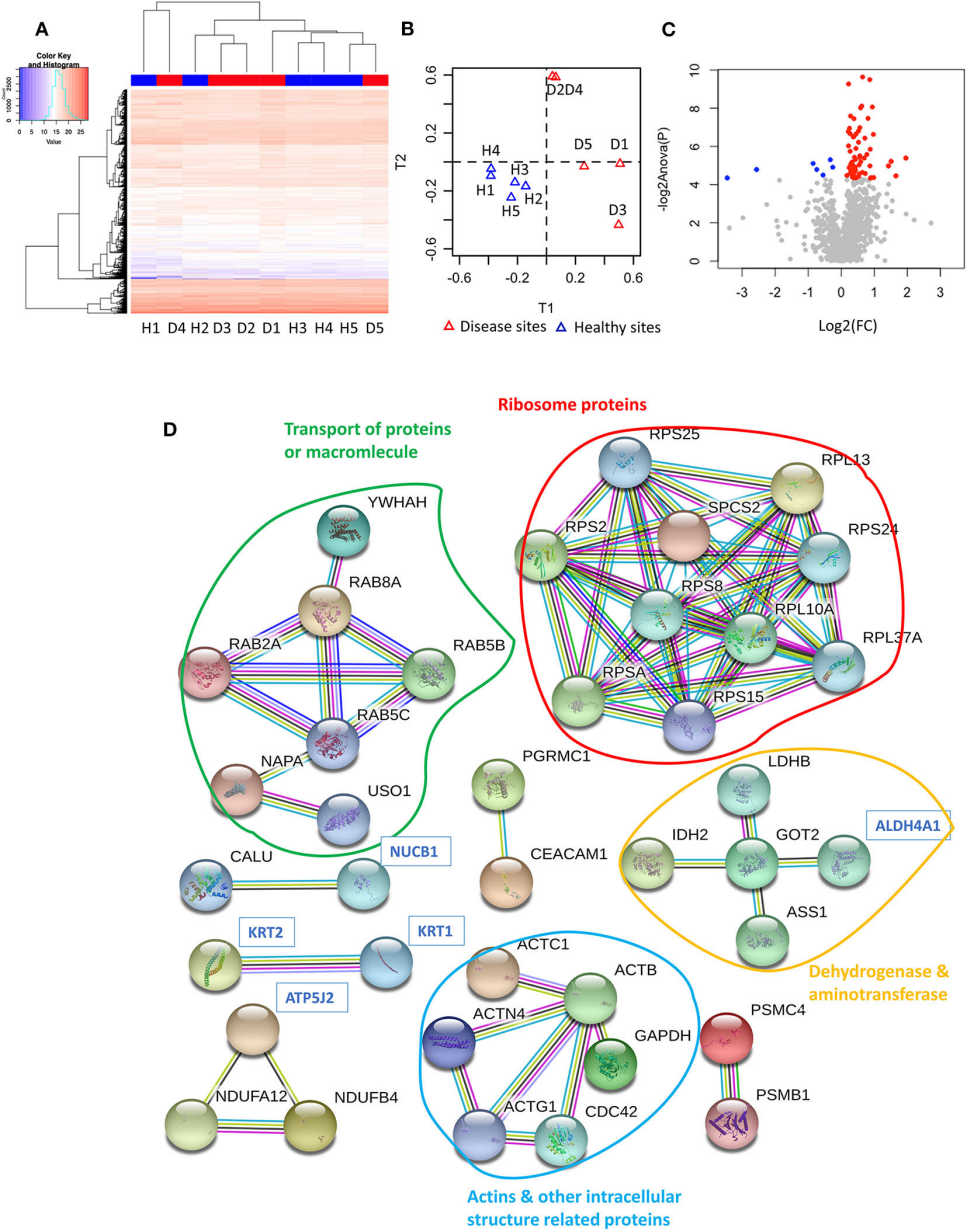
Proteome of gingival tissues from healthy and diseased sites. **(A)** The heatmap of normalised abundance for identified and quantified proteins in gingival tissue. Samples isolated from healthy sites were highlighted in blue colour, while samples isolated from diseased sites were highlighted in red. **(B)** The sPLS-DA plots represented the normalised abundance of all the 1,366 proteins from healthy (blue triangles) or diseased sites (red triangles). **(C)** 62 proteins were upregulated [log2 (FC) ≥ 0, *P* ≤ 0.05] in the disease compared with healthy sites (red dots), and 7 were downregulated [log2 (FC) ≤ 0, *P* ≤ 0.05]. **(D)** String analysis for the interaction between regulated proteins. Network established using STRING with interaction confident scores more than 0.9. The upregulated proteins were labelled in black, while the downregulated proteins were labelled in blue. The methods for acquiring the different protein-protein interactions were illustrated by different lines. The interaction confirmed by the curated database and experimental results were shown in blue and purple line, respectively. The interaction predicated by gene neighborhood, gene fusions, and gene co-occurrence were shown in green, red, and dark blue lines, respectively. While interaction was determined by text-mining, co-expression and protein homology were shown in yellow, black, and light blue lines, respectively. Healthy sites: H. Diseased sites: D.

Following a PCT-assisted label-free quantification work-flow, we obtained an overview of the gingiva proteome of 1,369 proteins (including 2 contaminant and 3 decoy proteins), with a protein false discovery rate (FDR) of 0.22%. Each quantified protein consisted of at least two unique peptides identified and quantified (Appendix Table 1). Although unsupervised hierarchical clustering analysis of the tissue proteomes could not distinguish healthy from diseased sites based on their normalised abundances (Figure 1A), this became possible by the utilisation of sPLS-DA (Figure 1B). Considering that protein regulation among individuals may vary, we assessed the differentially expressed protein levels by comparing intra-individually healthy and diseased sites, using paired *t*-test. Of all quantified proteins, 62 qualified as higher [log2 (FC) ≥ 0, *P* ≤ 0.05], whereas only 7 qualified as lower [log2 (FC) ≤ 0, *P* ≤ 0.05] in diseased sites compared to the respective healthy sites (Figure 1C, Appendix Table 2).

### Gene Ontology (GO) Analysis of the Regulated Proteins in the Gingival Tissue

The GO functions of differentially expressed proteins were annotated using the METACORE online software (https://portal.genego.com, Thomson Reuters). The top enriched GO terms for localisations, processes and molecular functions of all 69 regulated proteins were recognised and ranked according to their statistical significance (Table 1, Appendix Table 3). The major cellular localisation of the regulated proteins was “extracellular”-or “vesicle”-related (Table 1A). For instance, “extracellular exosome,” “extracellular vesicle,” and “extracellular organelle” were the top three enriched terms (Table 1A). Many of these proteins were linked to “localisation” or “transport” processes (e.g., “cellular localisation,” intracellular protein transport) (Table 1B), whereas their top molecular functions belonged to the “structural constituent of ribosome” and “structural molecule activity” (Table 1C).

**Table 1:** Enriched GO terms of regulated proteins.

**A: Top 10 enriched GO localisation of regulated proteins**			
#	**GO terms (Localisations)**	**Regulated**	***P*****-value**
1	Extracellular exosome	47/2,932	2.8778E-25
2	Extracellular vesicle	47/2,951	3.807E-25
3	Extracellular organelle	47/2,963	4.5382E-25
4	Extracellular space	49/4,428	1.7958E-19
5	Extracellular region part	50/4,693	2.8805E-19
6	Vesicle	50/4,975	3.6834E-18
7	Extracellular region	52/5,860	1.0373E-16
8	Intracellular organelle part	62/10,709	7.1559E-12
9	Organelle part	63/11,087	8.3233E-12
10	Cytoplasmic part	64/11,535	1.2786E-11
**B: Top 10 enriched GO processes of regulated proteins**			
#	**GO terms (Processes)**	**Regulated**	***P*****-value**
1	Establishment of localisation in the cell	40/2,418	5.532E-19
2	Cellular component organisation or biogenesis	63/7,803	2.3678E-16
3	Cellular localisation	41/3,189	1.3431E-15
4	Intracellular protein transport	27/1,234	2.5142E-15
5	Cellular protein localisation	33/2,036	4.6355E-15
6	Cellular component biogenesis	44/3,862	5.2894E-15
7	Cellular macromolecule localisation	33/2,048	5.4923E-15
8	Intracellular transport	33/2,068	7.2675E-15
9	Supramolecular fiber organisation	20/636	2.7295E-14
10	Establishment of protein localisation	32/2,036	3.3676E-14
**C: Top 10 enriched GO molecular functions of regulated proteins**			
**#**	**GO terms (Molecular functions)**	**Regulated**	***P*****-value**
1	Structural constituent of ribosome	9/202	1.4028E-08
2	Structural molecule activity	17/1,044	1.6659E-08
3	Cytoskeletal protein binding	16/1,100	2.2308E-07
4	Cadherin binding	9/335	1.0296E-06
5	Protein binding	62/14,119	1.3072E-06
6	RNA binding	19/1,919	4.6905E-06
7	Heterocyclic compound binding	40/6,982	6.5837E-06
8	Cell adhesion molecule binding	10/558	9.2596E-06
9	Organic cyclic compound binding	40/7,084	9.7525E-06
10	Actin binding	9/483	1.9857E-05

To further understand their inter-relationships, the protein-protein interactions were analysed using STRING (https://string-db.org/) (Appendix Table 4). When applying the highest confidence score (0.9), 40 among the 69 regulated proteins, formed 73 pairs of known such interactions, as illustrated by string networks (Figure 1D). The largest cluster of protein interactions consisted of 10 different proteins, which were mainly ribosomal ones. The second-largest cluster identified consisted of 8 proteins with assigned macromolecular transport properties (e.g., Ras-related proteins, general vesicular transport factor p115). In addition, among the interactions illustrated in the network, some were identified between actin and other intracellular-structural proteins, or between five dehydrogenases and aminotransferases, as well as more sparse interactions of only two or three proteins.

### Microbiome Profiles of Gingival Tissue Samples Cluster Based on Clinical State

**Figure 2:**
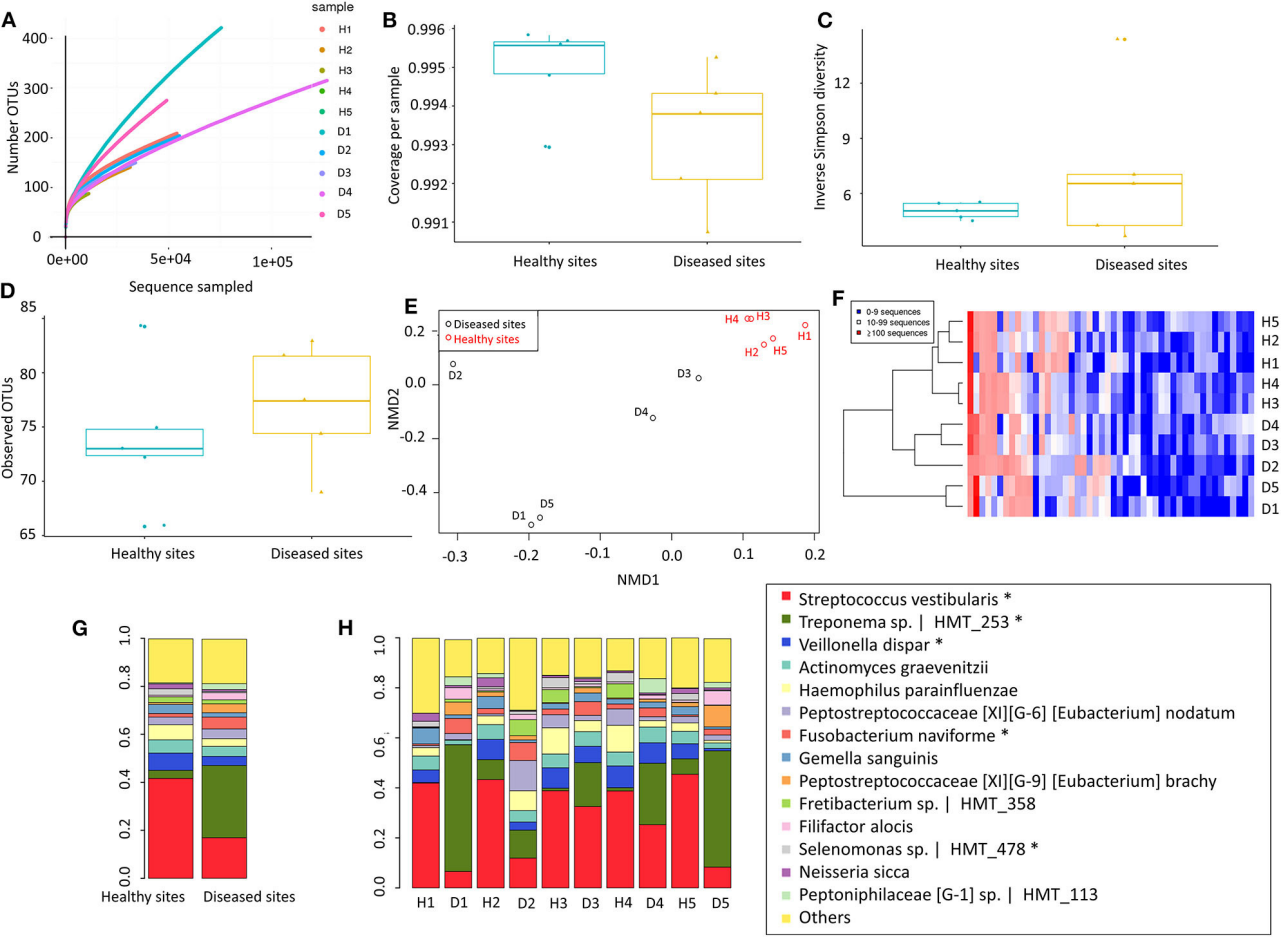
Microbiome of gingival tissues from healthy and diseased sites. **(A)** Rarefaction curve for the number of OTUs as a function of sampling effort. **(B)** Sample coverage. **(C)** Inverse Simpson diversity. **(D)** Observed OTU per group. **(E)** Non-metric multidimensional scaling (NMDS) for distance matrix of between disease and healthy samples. **(F)** OTU abundance across samples_ Abundant OTUs only (after removing rare OTUs). The bacterial relative abundance of the gingival tissue in **(G)** all healthy and disease tissues, or in **(H)** individual gingival tissues. The abundance that significant shift (*P* < 0.05) between two sites were highlighted in “*”. Healthy sites: H. Diseased sites: D.

Our next approach was to examine microorganisms present in the gingival tissues by establishing a microbial catalogue from 450,668 sequences that binned within 97% sequence identity from all ten-tissue samples (Appendix Table 5). On average, more than 119609.8 reads were identified from each sample, with a standard deviation of 77658.61. To analyse the alpha diversity, rarefaction curves were plotted based on the observed OTUs (Figure 2A), with calculated coverages for disease and health of 99.32 and 99.50%, respectively (Figure 2B), while the inverse Simpson diversity for disease and health were 7.18 and 5.05, respectively (Figure 2C). Furthermore, 362 non-rare OTUs were discovered among all ten-samples (data are available via ENA), with no significant differences in the number of detected OTUs between disease and health (*P* = 0.277, 77, and 74 average OTUs for diseased and healthy sites, respectively) using paired *t*-test (Figure 2D). To visualise differences in community structure between the groups, an NMDS plot of the thetayc distance was generated (Figure 2E), yielding sample clustering based on sites (healthy tissue vs. diseased tissue), not by sample pairing. Such sample clustering indicated the presence of different OTUs between diseased and healthy tissues. In addition, unsupervised hierarchical clustering analysis of OTU abundance also pointed to global microbiome differences between the two types of gingival tissues, where samples were also clustered according to tissue type (Figure 2F).

Only 14 species comprised more than 80% of 16S rRNA gene reads (Figure 2G). In healthy sites, *Streptococcus vestibularis* was the most abundant species, followed by *Haemophilus parahaemolyticus* and *Veillonella dispar* (Appendix Table 6). For diseased sites, the most abundant species was an as-yet-uncultured species *Treponema s*p. Human Microbial Taxon (HMT) 253, whereas the abundance of *S. vestibularis* declined from more than 20% in health sites, to <10% in diseased sites (Figure 2H). Eleven OTUs were significantly different (*P* < 0.05) between healthy and diseased sites, including five from the 14 most abundant species (i.e., *S. vestibularis, Treponema* sp. HMT 253*, V. dispar, Fusobacterium naviforme*, and *Selenomonas* sp. HMT 478) (Figure 2G).

### Gingival Tissue Interactome: Correlations Between Proteomes and Microbiomes of Gingival Tissues

**Figure 3:**
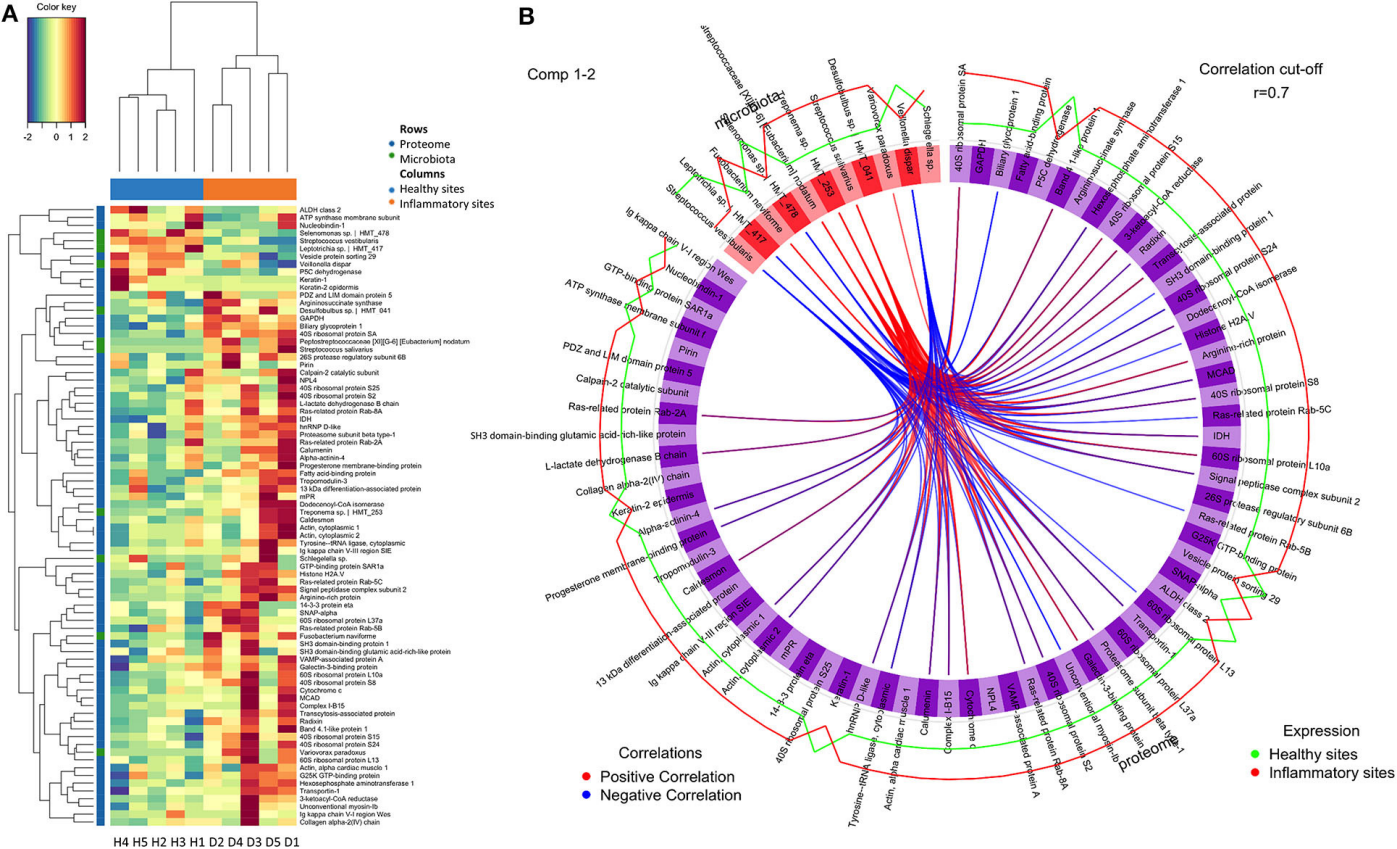
Correlations between regulated proteins and microbiotas. **(A)** The colour-coded clustered image map that present correlation between regulated proteins and microbiotas following pair-wise variable associations for canonical correlation analysis. **(B)** The Circos Plot showed variable correlations among regulated proteins and microbiotas (Appendix Table 7). Positive correlations were indicated with red lines, while negative correlations were indicated with blue lines. Only correlations more than 0.7 were showed. The levels of expression in healthy sites were indicated in green line, while the levels of expression in inflammatory sites were indicated in red. Healthy sites: H. Diseased sites: D.

To adequately address the interactome of the gingival tissues, potential correlations between the 69 regulated proteins and 11 regulated species were further analysed using the mixOmics package (Figure 3, Appendix Table 7). We found *S. vestibularis, V. dispar, Leptotrichia* sp. HMT_417, and *Selenomonas* sp. HMT_478 were clustered with all 7 downregulated proteins (i.e., Nucleobindin-1, Delta-1-pyrroline-5-carboxylate dehydrogenase, ATP synthase subunit f, Aldehyde dehydrogenase class 2, Keratin, type II cytoskeletal 1, Vacuolar protein sorting-associated protein 29, and Cytokeratin-2e), and negatively correlated with the 62 upregulated proteins (Figure 3A). On the contrary, five regulated species (more abundant in diseased sites), namely *Treponema* sp. HMT_253, *Streprococcus salivarius, Peptostreptococcaceae [XI][G-6] [Eubacterium] nodatum, Variovorax paradoxus* and *F. naviforme*, were strongly associated (*r* > 0.7) with 28, 29, 20, 1, and 4 upregulated proteins, respectively (Figure 3B, Appendix Table 7).

## Discussion

Different proteome and microbiome studies have been performed to understand periodontal diseases, yet few have focused on gingival tissues. Earlier studies indicated that the microbial content of the periodontal pocket in non-human primates or human determines the gene expression patterns in the gingival tissues (Papapanou et al., 2009; Ebersole et al., 2020). In the present study, we successfully applied a contemporary PCT-assisted workflow to dissect the gingival proteome and microbiome of both diseased and healthy sites from patients with periodontitis. The tissue recipient sites included both maxilla and mandible, which may exhibit different degrees of keratinisation. It should also be acknowledged that the stringent requirement of obtaining both a healthy and a diseased tissue specimen from the same donor prohibited us from limiting further the sampling criteria to either jaw, else it would have been more cumbersome to identify suitable patients.

The study also presents the most comprehensive quantitative proteome map of human gingival tissue to date, by quantifying 1,366 proteins, while earlier proteome analyses quantified <50 proteins at a time. Interestingly, only 69 of over 1,300 quantified proteins were significantly differentially expressed, whereas many of the inflammation-related proteins, such as Ig gamma-1 chain C region and Protein S100-A9, were abundantly expressed at both sites. The fact that the global proteomic profiles were similar between healthy and diseased sites denotes that protein composition of clinically healthy gingival tissue in the periodontal patient may have already been altered, even in the absence of evident clinical signs of inflammation at those sites. Of note, we used a data-dependent acquisition (DDA) strategy in this study. DDA only samples a subset of the most abundant ions detected during the first MS scan for the further fragmentation and sequential MS scans, while discards the rest. Hence, many proteins with demonstrated roles in periodontitis, including various cytokines, might have been masked due to their lower abundance. The high biological variabilities among patients may also contribute to low number of differentially expressed proteins. Nevertheless, this observation on overall protein regulations is consistent with transcriptomic changes observed in gingival tissue in periodontitis (Kebschull and Papapanou, 2011). Furthermore, the prediction of the protein functions shows that most of the regulated proteins were localised in the extracellular space. Similarly, increased numbers of secreted proteins were previously identified in human experimental gingivitis (Bostanci et al., 2013) and murine ligature-induced periodontitis (Bao et al., 2019). Hence, it is not surprising to find that many of the regulated proteins had transport-related functions. We also observed increases in the ribosome-related proteins (e.g., 60S ribosomal protein L37a, 40S ribosomal protein S25, 40S ribosomal protein S8, etc.), indicating ribosomal biosynthetic activity in the inflamed sites (Zhou et al., 2015).

Under homeostatic conditions, the host is in a balanced relationship with commensal oral species or potential pathobionts (Hajishengallis and Lamont, 2014). Hence, the concomitant study of the proteome and the microbiome is high relevant. One the microbiome aspect, 14 species accounted for 80% of the abundances, only 11 significantly differentiated between health and disease, six of which belonging to the rarely abundant group. This denotes that both high and low abundance species are to be considered in the future for defining signatures of target organisms capable of distinguishing between clinical health and disease. The oral microbiome data obtained in this study from gingival tissues identified potentially invasive species of the periodontium. Nevertheless, it cannot be definitively confirmed that the detected bacteria were all actual tissue invaders, and not superficial persisters after the washing steps. Yet, only a limited portion of the tissue interface has been in direct contact with the biofilm, which is dispersed during the homogenisation process of the specimen, thus down-playing the representation of the non-invaded species. Although their precise effects on the tissue may currently be unclear, different species were earlier found to co-exist within the gingival tissue (Baek et al., 2018). The relative abundances of different taxa may denote their potential roles in health and disease, or their invasive capacity. *Treponema* sp. HMT 253 and *F. naviforme* were significantly increased in diseased compared to healthy sites. For *Treponema s*p. HMT253, the only information currently available on this as-yet-uncultured species is its 16S rRNA gene clone library, derived from dental plaque of subjects with periodontitis and acute necrotising ulcerative gingivitis (Dewhirst et al., 2000). It is closely related to *Treponema denticola*, a potential pathogen implicated in periodontal disease (Dewhirst et al., 2010). Different models have shown that *T. denticola* is able to invade the epithelium and basement membrane (Grenier et al., 1990; Lux et al., 2001; Chi et al., 2003), as well as to secrete a chymotrypsin-like protease that can digest host components including type IV collagen, laminin and fibronectin (Grenier et al., 1990). *T. denticola* dentilisin was also reported to disrupt the epithelial cell monolayer (Chi et al., 2003). *Fusobacterium naviforme* (formerly F. nucleatum ssp. naviforme) has been identified and isolated from subgingival plaque samples (Colombo et al., 2009). Based on phylogenetic analysis (Dewhirst et al., 2010), it is expected to display functional similarities *F. nucleatum*. Although *Treponema* sp. HMT 253 and *F. naviforme* were not usually found as an abundant constituent of the subgingival biofilm in patients with periodontitis, their abundance within the tissue documented in this study suggests a greater invasion potential than their cultivated and characterised relatives. Of further note, other species that were elevated in diseased compared to healthy tissue, but did not reach statistical significance, are worth mentioning. Such were *F. alocis* and *Fretibacterium* sp. HMT 358, suggesting that they are potentially invasive of the gingival tissues. Both *F. alocis* and *Fretibacterium* sp. have been increasingly associated with periodontal disease. *Fretibacterium* sp. belongs to the phylum *Synergistetes* and is shown to be increased in the saliva of patients with periodontitis (Belibasakis et al., 2013) and in dental biofilms of patients with ANUG (Baumgartner et al., 2012), an invasive form of periodontal disease.

Some potentially invasive but generally less pathogenic species, including *S. vestibularis, V. dispar*, and *Selenomonas* sp. HMT 478, were enriched in healthy sites. Even though originally identified in the oral cavity (Whiley and Hardie, 1988), the presence of *S. vestibularis* has not been reported in periodontitis, but in other infectious diseases (Duan et al., 2017; Yilmaz et al., 2017). *V. dispar* is found in subgingival plaque from chronic periodontitis patients (Moon et al., 2015) and plays an import role when saliva is the main nutritional source of oral biofilm (Kolenbrander, 2011). The presence of *Selenomonas* spp. are reported in the salivary (Duan et al., 2017) or dental plaque microbiome (Paster et al., 2001; Faveri et al., 2008) of periodontal patients, but at a lower prevalence compared with other putative pathogens (Goncalves et al., 2012). Previous studies have shown that *Streptococcus* (Teles et al., 2012), *Veillonella* (Kolenbrander, 2011) and *Selenomonas* (Goncalves et al., 2012) contribute to the structural organisation of oral biofilm. *Streptococcus* spp. have the potential to colonise or invade the gingival tissue, but with no known association to gingival inflammation, which is well in line with our findings. It should be noted that, although diseased sites showed higher abundances of *Treponema* sp. HTM 253, most healthy sites fostered a fairly high proportion of this species. Perhaps this species allows other less invasive microorganisms like *Veillonella* spp. and *Streptococcus* spp. to penetrate the tissue barrier.

Thus far there has been inconsistent evidence to support or exclude the invasive properties of oral species in the pathogenesis of periodontal disease (Mendes et al., 2015). From an epidemiological perspective, there is sufficient evidence on the role of specific species as etiological agents of periodontitis, but the disease may be better understood as dysbiotic inflammation resulting from the concerted interaction of correlations (Lopez et al., 2015; Hajishengallis and Lamont, 2016). The interactome analysis of the present study indicates that groups of significantly elevated species and proteins tend to correlate with one another in health or disease. Information derived from such studies may decipher biological signatures in periodontal disease, which will help us understand its etiopathogenesis on the tissue level and may confer future diagnostic and prognostic value (Belibasakis and Mylonakis, 2015).

## Materials and Methods

### Study Population and Design

Gingival tissues (*n* = 10) were collected from two sites (one healthy and one diseased) of each five systematically healthy individuals with stage III periodontitis (age range from 45 to 56 years with a mean age 51.6 ± 4.5 years, F:M: 2:3). The study was approved by the Ethics Committee of Ege University (number 17–11.1/34) and conducted following the guidelines of the World Medical Association Declaration of Helsinki. Patients first attended the Department of Oral Diagnosis and Radiology for the completion of clinical and radiological examination procedures and were then directed to the specialised within the University Dental Clinics, for further assessment and treatment. Exclusion criteria were the use of tobacco products, presence of cardiovascular and respiratory diseases, diabetes mellitus, HIV infection, systemic inflammatory conditions or non-plaque-induced oral inflammatory conditions, immunosuppressive chemotherapy, and current pregnancy or lactation. None of the patients had a history of periodontal therapy or had taken medication such as antibiotics or anti-inflammatory drugs that could affect their periodontal status for at least 6 months prior to the study. Informed consent was obtained from all participants. Full-mouth and site-specific periodontal parameters including PPD, CAL, dichotomous presence of BOP, and plaque for each patient were recorded. The full-mouth means PPD (mm) and CAL (mm) were 5.1 ± 0.4, 5.7 ± 0.4, respectively. The full-mouth mean plaque and BOP scores were 72.0 ± 6.7 and 75.0 ± 5.0, respectively.

### Collection of Gingival Tissue Samples

**Figure 4:**
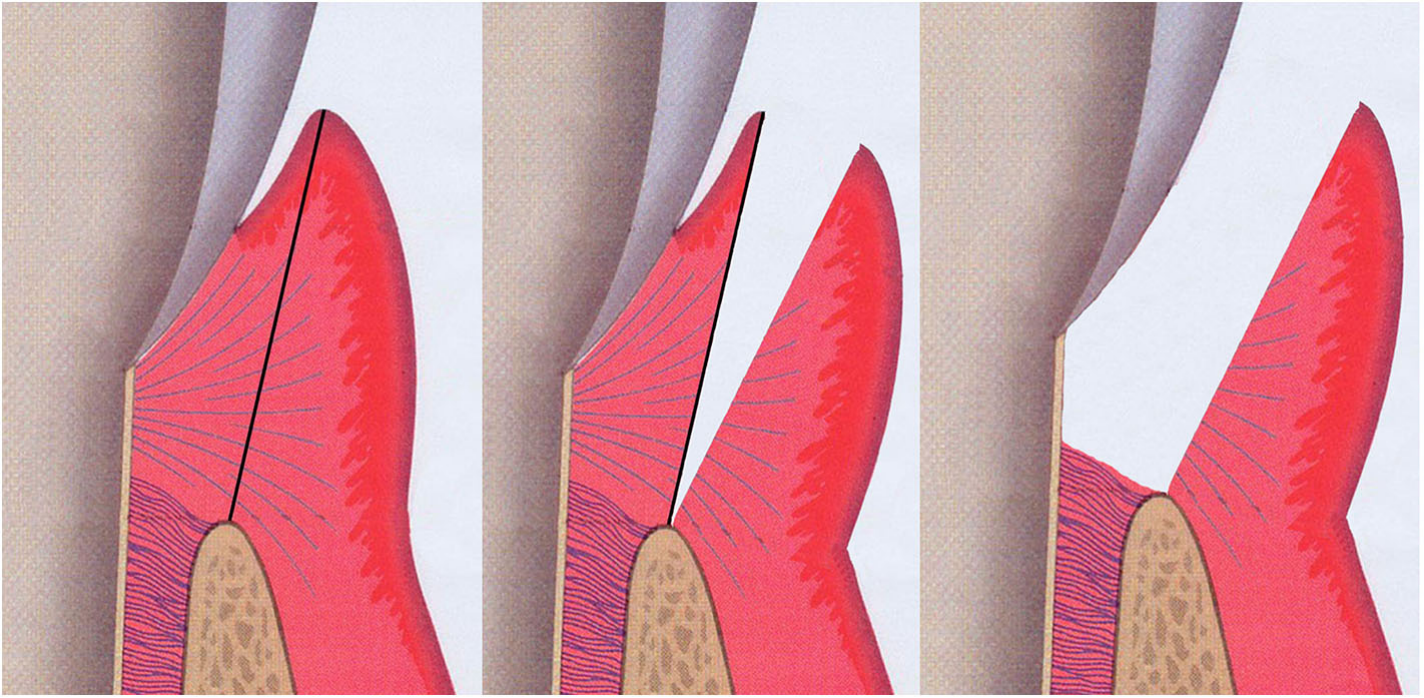
Illustration of the sample sits for gingival tissue. Both pocket epithelium and underlying connective tissue were included in the gingival specimen taken from the approximal sites of the selected teeth (grey; tooth, pink; gingival connective tissue, purple-red; gingival epithelium, brown-beige; alveolar bone).

Gingival tissue samples, including both pocket epithelium and underlying connective tissue, were taken from the approximal sites of the selected teeth prior to non-surgical periodontal therapy (Figure 4). The “healthy sites” had no clinical signs of gingival inflammation (no BOP), exhibited a PPD of ≤ 3 mm and had no radiographic evidence of alveolar bone loss and no CAL. These healthy tissues were sampled when the premolars were scheduled to have periodontal crown lengthening surgery. The “diseased sites” showed BOP, had an interproximal PPD of ≥6 mm, and a concomitant CAL of ≥6 mm. Two gingival tissue samples from each participant were obtained and washed with sterile normal saline solution to remove any blood or detached biofilms on the tissue surface. Tissues were then placed in a sterile tube containing a tissue protectant solution (RNAlater, Sigma-Aldrich) and stored at +4°C overnight, before long-term storage at −70°C until later usage.

### Protein Extraction and Digestion

Gingival tissues were washed three times, each for 5 min in PBS to remove any residues prior to lysis. The tissues were then lysed and digested using a Barocycler NEP2320 (Pressure BioSciences) at 33°C as described previously (Bao et al., 2019). In brief, 2.5 to 3 mg of samples (*n* = 10) were placed in MicroTubes (Pressure BioSciences) and lysed with a 60–cycle barocyling process. The exacted proteins were then reduced and alkylated using tris (2-carboxyethyl) phosphine (Sigma) and iodoacetamide (Sigma). Later, extracted proteins were digested using Lys-C (Wako) at an enzyme-to-protein (estimated to be 10% of the wet sample weight) ratio of a 1:45 with a 45–cycle barocyling process. These resultant solutions were further diluted and then digested again using trypsin (Promega) at an enzyme-to-protein ratio of 1:50 with a 90-cycle process. Each barocyling-cycle mentioned above consisted of a 50 s ultra-high pressure phase (45, 20, 20 thousand pounds per square inch (KPSI) for 60-, 45-, 90-cycle process, respectively) followed with a 10 s ambient pressure phase in each cycle. Resultant solutions were acidified by trifluoroacetic acid (TFA) (Sigma) to a final concentration of 0.8% w/v, desalted using reverse-phase cartridges Finisterre SPE C18 (Wicom International AG), dried with vacuum centrifuged, and kept in −20°C freezer until further use.

### Mass Spectrometry (MS) and Data Analysis

All frozen peptides were reconstituted in 3% acetonitrile ACN in 0.1% formic acid and adjusted to 0.5 μg/μl using NanoDrop 1000 spectrophotometer (WITEC AG). One microlitre of desalted peptide was analysed on an Orbitrap Fusion mass spectrometer (Thermo Fisher Scientific) for proteomic analysis as described previously (Bao et al., 2017).

Label-free quantification was performed by Progenesis QI for proteomics (Non-linear Dynamics) as described previously (Bao et al., 2017). In brief, .raw files of individual samples were aligned with a pooled sample to create a Mascot files (.mgf). This .mgf files was searched with Mascot (version 2.4.1, Matrix Science) using the following search parameters: precursor tolerance: ± 10 ppm; fragment ion tolerance: ± 0.6 Da; enzyme: trypsin; maximum missed cleavages: 2; fixed medication: carbamidomethylation of cysteine; variable modification: deamidated (NQ), oxidation (M) and acetylation on protein N-termini. Data were searched against a FASTA file (40,510 sequences and 22,667,481 residues), consisting of the human proteome from UniProt (isoforms included, retrieved December 9th 2016), contaminant database from the FGCZ, the resulting.dat file was imported into Scaffold v4.0 (Proteome Software) to generate spectrum report, with protein false discovery rate (FDR) of 10%, minimal one peptide and peptide FDR of 5%. Finally, the spectrum report was imported back into Progenesis QI for identifying the quantified proteins. The “within-subject” option was used for experiment design set up. Proteins with a minimum of two unique quantified peptides and a significant ANOVA *p*-value smaller than 0.05 were considered as differentially regulated ones.

### Data Clustering and Heat Maps for Regulated Proteins

Unsupervised clustering analysis and heat maps of regulated proteins were generated using the R software (R: A Language and Environment for Statistical Computing, R Development Core Team) in particular the Quantable packages (https://cran.r-project.org/web/packages/quantable/index.html) to obtain a global visualisation and regulation trends of protein profiles. Apparent outliers were excluded from the quantification. Sparse Partial Least Squares Discriminant Analysis (sPLS-DA) was used to visualise the similarity between healthy and inflammation sites using the SPLS package (Chun and Keles, 2010).

### Functional Analysis of the Regulated Proteins

The regulated proteins were subjected to Metacore online database (29th May 2019) for “gene ontology (GO) enrichment analysis.” Enriched GO terms were recognised and ranked according to their statistical significance (–log2P value), using a hypergeometric distribution.

### Sample Disruption and DNA Extraction

Other gingival tissues (approximal 2.5 to 3 mg, *n* = 10) were lysed using a Barocycler NEP2320 (Pressure BioSciences) with only a 60–cycle barocyling process (each consisting 50 s at 45 KPSI followed by 10 s at ambient pressure) at 33°C for DNA extraction. The genomic DNA from lysates was extracted using the GenElute™ Bacterial Genomic DNA Kit (Sigma) and stored at −20°C until further use.

### Sample Preparation for 16S rRNA Gene Sequencing

The hypervariable regions 7 to 9 (V7-9) of the 16S rRNA gene were amplified in the first round of PCR from isolated genomic DNA using universal bacterial primers that also contained the Illumina Truseq primer binding site (Appendix Table 8). Amplification reactions were performed in a total volume of 25 μl containing 5X KAPA HiFi Buffer, 10 mM KAPA dNTP Mix, 0.5 U KAPA HiFi DNA Polymerase (KAPA Biosystems), 4 μM of primers ordered from Microsynth (Balgach), and 22.4 ng DNA diluted in DNA-free water. The PCR amplification was performed on a Verity thermocycler (Thermo Fisher Scientific) with the following cycling conditions: 95°C for 5 min, 25 cycles at 98°C each for 20 s, 70°C for 30 s, 72°C for 30 s and a final extension at 72°C for 10 min. The PCR reactions were run on a 2% agarose gel, the amplicon band was cut and extracted using MinElute Gel Extraction Kit (Qiagen) and eluted in 50 μl DNase-free water.

In the second round of PCR, the remaining Illumina Truseq adaptors together with dual indexing Truseq barcodes were incorporated into the previously amplified material (Appendix Table 9). Amplification reactions were performed in a total volume of 50 μl containing 5X KAPA HiFi Buffer, 10 mM KAPA dNTP Mix, 1 U KAPA HiFi DNA Polymerase (KAPA Biosystems), 4 μM of the primers ordered from Microsynth (Balgach) and 22.5 ng of the previously amplified material diluted in DNA-free water. PCR amplification was performed on a Verity thermocycler (Thermo Fisher Scientific) with the following cycling conditions: 98°C for 5 min, five cycles at 98°C each for 30 s, 54°C for 30 s, 72°C for 30 s and a final extension at 72°C for 5 min. PCR products were gel-purified and eluted in 50 μl DNase-free water. The quality and quantity of resulting amplicon libraries were validated using Qubit® (1.0, Invitrogen) Fluorometer and the Tapestation **(**Agilent). The amplicons from the different samples were normalised to 4 nM in Tris-Cl 10 mM, pH8.5 with 0.1% Tween 20 and as they contain dual indexes, they were equimolarly pooled and paired-end sequenced in a Miseq Illumina Instrument (Illumina CA) using a 600cycle V3 kit.

### Processing and Taxonomic Classification of 16S rRNA Gene Reads

MiSeq paired-end (PE) reads were first filtered based on average quality (>= Q20) using Trimmomatic (version 0.36) (Bolger et al., 2014). Quality checked PE reads were processed using mothur (version 1.38.1) (Schloss et al., 2009), following the MiSeq SOP (Standard Operation Protocol) (https://www.mothur.org/wiki/MiSeq_SOP). In detail, the quality-filtered PE reads were joined into contig sequences. Identical sequences were merged and the counts of all unique sequences were recorded. Unique sequences were aligned guided by the Silva bacterial 16S reference alignment (Release 102) (Quast et al., 2013). After alignment, the bulk of the sequences started at position 34,476 and ended at position 43,116 of the reference alignment. Sequences aligned at the different start and/or stop sites, as well as sequences with homopolymers longer than 8 nt were filtered out. Sites containing only gap characters were also removed. Sequences were pre-clustered allowing for up to three base differences. Chimaera sequences were removed using the UCHIME algorithm (Edgar et al., 2011). Sequences were initially classified by comparing them to the mothur-formatted RDP training set (v.9), with cutoff values set at genus level (Cole et al., 2014). This taxonomic information was used to remove undesired contaminants (Chloroplast, Mitochondria, unknown Archaea and Eukaryota) and to split the sequences into 16S genus bins (taxlevel=6) and one un-classified bin. Each of the 166 bins was then clustered into operational taxonomic units (OTUs) using the single-linkage clustering algorithm implemented in hpc-clust (Matias Rodrigues and von Mering, 2014), with 97% sequence similarity as the cutoff. The mothur-compatible OTU list was prepared using the utility script “makeotus_mothur” in the same software and imported into mothur for OTU-based analysis. To taxonomically classify OTUs, representative sequences (the most abundant) were compared against the Human Oral Microbiome Database (HOMD) (Dewhirst et al., 2010) using BLASTN in ncbi-blast-2.6.0+ (Altschul et al., 1990). The taxonomy of the best match (with >96.99% homology) was assigned to the corresponding OTU. If the representative sequence had <97% homology to the HOMD reference, the genus name was used to taxonomically designate the OTU.

### Microbiome Data Analysis

In all analysis where normalisation was applied, standardised datasets were generated by randomly selecting 3,597 sequences 1,000 times from each sample. To analyse the alpha diversity of the samples, rarefaction curves describing the number of OTUs observed as a function of sampling effort were plotted. The numbers of sequences, the sample coverage, the number of observed OTUs, and the Inverse Simpson diversity were calculated. To compare the membership and structure of the samples between groups, distance matrices for the classical Jaccard (1908) and Yue and Clayton theta values (Yue and Clayton, 2005) were calculated. The distance matrices were also visualised using NMDS (non-metric multidimensional scaling) (Clarke, 1993). The R software, in particular, the packages “gplots” and “stats,” were used to generate unsupervised clustering analysis and heatmaps of non-rare OTUs (abundance ≥ 50 across all samples). Differentially represented OTUs were evaluated via paired Student's *t*-test using their relative abundances *p* < 0.05. Benjamini-Hochberg corrected *P*-values and power calculations were provided for each OTUs.

### Correlations Between Microbiome and Protein Datasets

To visualise the correlation between differentially presented OTUs and proteins, Circos plots and cluster-imagine maps were generated for r values (Pair-wise variable associations for canonical correlation analysis correlation between variables, defined by a generalisation of the cosine angle between the center of the circle and each variable point; Gonzalez et al., 2012) using the mixOmics package in R (Rohart et al., 2017).

## Data Availability Statement

The datasets presented in this study can be found in online repositories. The names of the repository/repositories and accession number(s) can be found here: https://www.ebi.ac.uk/ena, PRJEB27125 (ERP109167); http://www.proteomexchange.org/, PXD018029.

## Ethics Statement

The studies involving human participants were reviewed and approved by Ethics Committee of Ege University (number 17-11.1/34). The participants provided their written informed consent to participate in this study.

## Author Contributions

KB contributed to data acquisition, analysis, interpretation, drafted, and critically revised the manuscript. XL, LP, and WQ contributed to data analysis and interpretation as well as critically revised the manuscript. NS, JG, PD, and GH critically revised the manuscript. PG and GE contributed to conception and design, data acquisition, and critically revised the manuscript. NB and GB contributed to conception and design, data interpretation, drafted, and critically revised the manuscript. All authors gave final approval and agreed to be accountable for all aspects of the work.

## Conflict of Interest

The authors declare that the research was conducted in the absence of any commercial or financial relationships that could be construed as a potential conflict of interest.

## References

[B1] Altschul S. F. Gish W. Miller W. Myers E. W. Lipman D. J. (1990). Basic local alignment search tool. J. Mol. Biol. 215, 403–410. 10.1016/S0022-2836(05)80360-2 2231712

[B2] Baek K. Ji S. Choi Y. (2018). Complex intratissue microbiota forms biofilms in periodontal lesions. J. Dent. Res. 97, 192–200. 10.1177/0022034517732754 28945499 PMC6429573

[B3] Bao K. Bostanci N. Thurnheer T. Belibasakis G. N. (2017). Proteomic shifts in multi-species oral biofilms caused by *Anaeroglobus geminatus*. Sci. Rep 7:4409. 10.1038/s41598-017-04594-9 28667274 PMC5493653

[B4] Bao K. Li X. Kajikawa T. Toshiharu A. Selevsek N. Grossmann J. (2019). Pressure cycling technology assisted mass spectrometric quantification of gingival tissue reveals proteome dynamics during the initiation and progression of inflammatory periodontal disease. Proteomics 2019:e1900253 10.1002/pmic.201900253 PMC7033018 31881116

[B5] Baumgartner A. Thurnheer T. Luthi-Schaller H. Gmur R. Belibasakis G. N. (2012). The phylum synergistetes in gingivitis and necrotizing ulcerative gingivitis. J. Med. Microbiol. 61(Pt. 11), 1600–1609. 10.1099/jmm.0.047456-0 22878253

[B6] Belibasakis G. N. Mylonakis E. (2015). Oral infections: clinical and biological perspectives. Virulence 6, 173–176. 10.1080/21505594.2015.1025191 25830413 PMC4601476

[B7] Belibasakis G. N. Ozturk V. O. Emingil G. Bostanci N. (2013). Synergistetes cluster A in saliva is associated with periodontitis. J. Periodont. Res. 48, 727–732. 10.1111/jre.12061 23441995

[B8] Bertoldi C. Bellei E. Pellacani C. Ferrari D. Lucchi A. Cuoghi A.. (2013). Non-bacterial protein expression in periodontal pockets by proteome analysis. J. Clin. Periodontol. 40, 573–582. 10.1111/jcpe.12050 23509886

[B9] Bolger A. M. Lohse M. Usadel B. (2014). Trimmomatic: a flexible trimmer for Illumina sequence data. Bioinformatics 30, 2114–2120. 10.1093/bioinformatics/btu170 24695404 PMC4103590

[B10] Bostanci N. Bao K. (2017). Contribution of proteomics to our understanding of periodontal inflammation. Proteomics 17:1500518. 10.1002/pmic.201500518 27995754

[B11] Bostanci N. Ramberg P. Wahlander A. Grossman J. Jonsson D. Barnes V. M.. (2013). Label-free quantitative proteomics reveals differentially regulated proteins in experimental gingivitis. J. Proteome Res. 12, 657–678. 10.1021/pr300761e 23244068

[B12] Chi B. Qi M. Kuramitsu H. K. (2003). Role of dentilisin in *Treponema denticola* epithelial cell layer penetration. Res. Microbiol. 154, 637–643. 10.1016/j.resmic.2003.08.001 14596901

[B13] Chun H. Keles S. (2010). Sparse partial least squares regression for simultaneous dimension reduction and variable selection. J. R. Stat. Soc. Series B Stat. Methodol. 72, 3–25. 10.1111/j.1467-9868.2009.00723.x 20107611 PMC2810828

[B14] Clarke K. R. (1993). Non-parametric multivariate analyses of changes in community structure. Austral J. Ecol. 18, 117–143. 10.1111/j.1442-9993.1993.tb00438.x

[B15] Cole J. R. Wang Q. Fish J. A. Chai B. McGarrell D. M. Sun Y.. (2014). Ribosomal database project: data and tools for high throughput rRNA analysis. Nucleic Acids Res. 42, D633–D642. 10.1093/nar/gkt1244 24288368 PMC3965039

[B16] Colombo A. P. Boches S. K. Cotton S. L. Goodson J. M. Kent R. Haffajee A. D.. (2009). Comparisons of subgingival microbial profiles of refractory periodontitis, severe periodontitis, and periodontal health using the human oral microbe identification microarray. J. Periodontol. 80, 1421–1432. 10.1902/jop.2009.090185 19722792 PMC3627366

[B17] Colombo A. V. da Silva C. M. Haffajee A. Colombo A. P. (2007). Identification of intracellular oral species within human crevicular epithelial cells from subjects with chronic periodontitis by fluorescence *in situ* hybridization. J. Periodont. Res. 42, 236–243. 10.1111/j.1600-0765.2006.00938.x 17451543

[B18] Courtois G. J. III. Cobb C. M. Killoy W. J. (1983). Acute necrotizing ulcerative gingivitis. A transmission electron microscope study. J. Periodontol. 54, 671–679. 10.1902/jop.1983.54.11.671 6580420

[B19] Demmer R. T. Behle J. H. Wolf D. L. Handfield M. Kebschull M. Celenti R.. (2008). Transcriptomes in healthy and diseased gingival tissues. J. Periodontol. 79, 2112–2124. 10.1902/jop.2008.080139 18980520 PMC2637651

[B20] Dewhirst F. E. Chen T. Izard J. Paster B. J. Tanner A. C. Yu W. H.. (2010). The human oral microbiome. J. Bacteriol. 192, 5002–5017. 10.1128/JB.00542-10 20656903 PMC2944498

[B21] Dewhirst F. E. Tamer M. A. Ericson R. E. Lau C. N. Levanos V. A. Boches S. K.. (2000). The diversity of periodontal spirochetes by 16S rRNA analysis. Oral Microbiol. Immunol. 15, 196–202. 10.1034/j.1399-302x.2000.150308.x 11154403

[B22] Duan X. Wu T. Xu X. Chen D. Mo A. Lei Y.. (2017). Smoking may lead to marginal bone loss around non-submerged implants during bone healing by altering salivary microbiome: a prospective study. J. Periodontol. 88, 1297–1308. 10.1902/jop.2017.160808 28844190

[B23] Ebersole J. Kirakodu S. Chen J. Nagarajan R. Gonzalez O. A. (2020). Oral microbiome and gingival transcriptome profiles of ligature-induced periodontitis. J. Dent. Res. 99, 746–757. 10.1177/0022034520906138 32075482 PMC7243415

[B24] Edgar R. C. Haas B. J. Clemente J. C. Quince C. Knight R. (2011). UCHIME improves sensitivity and speed of chimera detection. Bioinformatics 27, 2194–2200. 10.1093/bioinformatics/btr381 21700674 PMC3150044

[B25] Faveri M. Mayer M. P. Feres M. de Figueiredo L. C. Dewhirst F. E. Paster B. J. (2008). Microbiological diversity of generalized aggressive periodontitis by 16S rRNA clonal analysis. Oral Microbiol. Immunol. 23, 112–118. 10.1111/j.1399-302X.2007.00397.x 18279178

[B26] Goncalves L. F. Fermiano D. Feres M. Figueiredo L. C. Teles F. R. Mayer M. P.. (2012). Levels of *Selenomonas* species in generalized aggressive periodontitis. J. Periodont. Res. 47, 711–718. 10.1111/j.1600-0765.2012.01485.x 22612405 PMC3678906

[B27] Gonzalez I. Cao K. A. Davis M. J. Dejean S. (2012). Visualising associations between paired 'omics' data sets. BioData Min 5:19. 10.1186/1756-0381-5-19 23148523 PMC3630015

[B28] Grenier D. Uitto V. J. McBride B. C. (1990). Cellular location of a *Treponema denticola* chymotrypsinlike protease and importance of the protease in migration through the basement membrane. Infect. Immun. 58, 347–351. 10.1128/IAI.58.2.347-351.1990 2404867 PMC258461

[B29] Hajishengallis G. Lamont R. J. (2014). Breaking bad: manipulation of the host response by *Porphyromonas gingivalis*. Eur. J. Immunol. 44, 328–338. 10.1002/eji.201344202 24338806 PMC3925422

[B30] Hajishengallis G. Lamont R. J. (2016). Dancing with the stars: how choreographed bacterial interactions dictate nososymbiocity and give rise to keystone pathogens, accessory pathogens, and pathobionts. Trends Microbiol. 24, 477–489. 10.1016/j.tim.2016.02.010 26968354 PMC4874887

[B31] Jaccard P. (1908). Nouvelles recherches Sur La distribution florale. Bull. Soc. Vaudoise Sci. Nat. 44, 223–270.

[B32] Kebschull M. Papapanou P. N. (2011). Periodontal microbial complexes associated with specific cell and tissue responses. J. Clin. Periodontol. 38(Suppl. 11), 17–27. 10.1111/j.1600-051X.2010.01668.x 21323700

[B33] Kolenbrander P. E. (2011). Multispecies communities: interspecies interactions influence growth on saliva as sole nutritional source. Int. J. Oral Sci. 3, 49–54. 10.4248/IJOS11025 21485308 PMC3469885

[B34] Listgarten M. A. (1965). Electron microscopic observations on the bacterial flora of acute necrotizing ulcerative gingivitis. J. Periodontol. 36, 328–339. 10.1902/jop.1965.36.4.328 14326701

[B35] Lopez R. Hujoel P. Belibasakis G. N. (2015). On putative periodontal pathogens: an epidemiological perspective. Virulence 6, 249–257. 10.1080/21505594.2015.1014266 25874553 PMC4601192

[B36] Lux R. Miller J. N. Park N. H. Shi W. (2001). Motility and chemotaxis in tissue penetration of oral epithelial cell layers by Treponema denticola. Infect. Immun. 69, 6276–6283. 10.1128/IAI.69.10.6276-6283.2001 11553571 PMC98762

[B37] Matias Rodrigues J. F. von Mering C. (2014). HPC-CLUST: distributed hierarchical clustering for large sets of nucleotide sequences. Bioinformatics 30, 287–288. 10.1093/bioinformatics/btt657 24215029 PMC3892691

[B38] Mendes L. Azevedo N. F. Felino A. Pinto M. G. (2015). Relationship between invasion of the periodontium by periodontal pathogens and periodontal disease: a systematic review. Virulence 6, 208–215. 10.4161/21505594.2014.984566 25654367 PMC4601159

[B39] Monari E. Cuoghi A. Bellei E. Bergamini S. Lucchi A. Tomasi A.. (2015). Analysis of protein expression in periodontal pocket tissue: a preliminary study. Proteome Sci. 13:33. 10.1186/s12953-015-0089-y 26719749 PMC4696085

[B40] Moon J. H. Lee J. H. Lee J. Y. (2015). Subgingival microbiome in smokers and non-smokers in Korean chronic periodontitis patients. Mol. Oral Microbiol. 30, 227–241. 10.1111/omi.12086 25283067

[B41] Papapanou P. N. Behle J. H. Kebschull M. Celenti R. Wolf D. L. Handfield M.. (2009). Subgingival bacterial colonization profiles correlate with gingival tissue gene expression. BMC Microbiol 9:221. 10.1186/1471-2180-9-221 19835625 PMC2771036

[B42] Paster B. J. Boches S. K. Galvin J. L. Ericson R. E. Lau C. N. Levanos V. A.. (2001). Bacterial diversity in human subgingival plaque. J. Bacteriol. 183, 3770–3783. 10.1128/JB.183.12.3770-3783.2001 11371542 PMC95255

[B43] Quast C. Pruesse E. Yilmaz P. Gerken J. Schweer T. Yarza P.. (2013). The SILVA ribosomal RNA gene database project: improved data processing and web-based tools. Nucleic Acids Res. 41, D590–D596. 10.1093/nar/gks1219 23193283 PMC3531112

[B44] Rohart F. Gautier B. Singh A. Le Cao K. A. (2017). mixOmics: an R package for 'omics feature selection and multiple data integration. PLoS Comput. Biol. 13:e1005752. 10.1371/journal.pcbi.1005752 29099853 PMC5687754

[B45] Schloss P. D. Westcott S. L. Ryabin T. Hall J. R. Hartmann M. Hollister E. B.. (2009). Introducing mothur: open-source, platform-independent, community-supported software for describing and comparing microbial communities. Appl. Environ. Microbiol. 75, 7537–7541. 10.1128/AEM.01541-09 19801464 PMC2786419

[B46] Teles F. R. Teles R. P. Uzel N. G. Song X. Q. Torresyap G. Socransky S. S.. (2012). Early microbial succession in redeveloping dental biofilms in periodontal health and disease. J. Periodont. Res. 47, 95–104. 10.1111/j.1600-0765.2011.01409.x 21895662 PMC3253172

[B47] Wang J. Ma Z. Carr S. A. Mertins P. Zhang H. Zhang Z.. (2017). Proteome profiling outperforms transcriptome profiling for coexpression based gene function prediction. Mol. Cell. Proteomics 16, 121–134. 10.1074/mcp.M116.060301 27836980 PMC5217778

[B48] Whiley R. A. Hardie J. M. (1988). *Streptococcus vestibularis* sp. nov. from the human oral cavity. Int. J. Syst. Evol. Microbiol. 38:5 10.1099/00207713-38-4-335

[B49] Yaprak E. Kasap M. Akpinar G. Kayaalti-Yuksek S. Sinanoglu A. Guzel N.. (2018). The prominent proteins expressed in healthy gingiva: a pilot exploratory tissue proteomics study. Odontology 106, 19–28. 10.1007/s10266-017-0302-9 28382581

[B50] Yilmaz F. Bora F. Ersoy F. (2017). “*Streptococcus vestibularis*”: a rare cause of peritoneal dialysis-related peritonitis. Ther. Apher. Dial. 21, 418–419. 10.1111/1744-9987.12538 28321985

[B51] Yue J. C. Clayton M. K. (2005). A similarity measure based on species proportions. Commun. Stat. Theor. Methods 34, 2123–2131. 10.1080/STA-200066418

[B52] Zhou X. Liao W. J. Liao J. M. Liao P. Lu H. (2015). Ribosomal proteins: functions beyond the ribosome. J. Mol. Cell Biol. 7, 92–104. 10.1093/jmcb/mjv014 25735597 PMC4481666

